# Review of Herbal Traditional Chinese Medicine for the Treatment of Diabetic Nephropathy

**DOI:** 10.1155/2016/5749857

**Published:** 2015-11-15

**Authors:** Guang-dong Sun, Chao-yuan Li, Wen-peng Cui, Qiao-yan Guo, Chang-qing Dong, Hong-bin Zou, Shu-jun Liu, Wen-peng Dong, Li-ning Miao

**Affiliations:** Department of Nephrology, Second Hospital of Jilin University, Changchun 130041, China

## Abstract

Diabetic nephropathy (DN) is the most serious chronic complications of diabetes; 20–40% of diabetic patients develop into end stage renal disease (ESRD). However, exact pathogenesis of DN is not fully clear and we have great difficulties in curing DN; poor treatment of DN led to high chances of mortality worldwide. A lot of western medicines such as ACEI and ARB have been demonstrated to protect renal function of DN but are not enough to delay or retard the progression of DN; therefore, exploring exact and feasible drug is current research hotspot in medicine. Traditional Chinese medicine (TCM) has been widely used to treat and control diabetes and its complications such as DN in a lot of scientific researches, which will give insights into the mechanism of DN, but they are not enough to reveal all the details. In this paper, we summarize the applications of herbal TCM preparations, single herbal TCM, and/or monomers from herbal TCM in the treatment of DN in the recent 10 years, depicting the renal protective effects and the corresponding mechanism, through which we shed light on the renal protective roles of TCM in DN with a particular focus on the molecular basis of the effect and provide a beneficial supplement to the drug therapy for DN.

## 1. Introduction

Diabetic nephropathy (DN) is a widely recognized microvascular complication of diabetes and almost the leading cause of end-stage kidney failure worldwide responsible for morbidity and mortality [1]. Clinical manifestations of DN include initial increase in glomerular filtration (GFR), proteinuria, increased creatinine levels, and eventually decreased GFR [2–4]. Major pathological changes of DN are virtually indistinguishable in both type 1 and type 2 diabetes, including mesangial expansion, extracellular matrix (ECM) accumulations, tubulointerstitial fibrosis, and glomerular sclerosis. Hyaline arteriolosclerosis is often prominent in the established DN pathological features caused by endothelial dysfunction and inflammation [5–7].

Multiple factors have been implicated in the pathogenesis of DN including hyperglycemia induced activation of advanced glycation end products (AGEs) and reactive oxygen species (ROS); JAK-STAT pathways and G protein signaling; activation of the PKC, renin-angiotensin aldosterone system (RAAS), transforming growth factor *β*-Smad-mitogen-activated protein kinase (TGF-*β*-Smad-MAPK), deregulated expression of cyclin dependent kinases (CDK), and their inhibitors; and aberrant expression of ECM proteins, ECM-degrading enzymes, metalloproteinases, and their inhibitors [8]. The abovementioned factors can induce aberrant expression of profibrotic and proinflammatory cytokines, cell-cycle genes, and ECM genes involved in DN [9]. A large number of novel treatment options has arisen from experimental studies based on the pathogenic factors of DN, including intensive glycemic control, precise blood pressure control, optimal RAAS blockade with ACEI/ARB, life style modifications such as exercise and dietary restrictions, and a lot of novel agents [10], but the portion of ESRD due to DN still remains high in spite of the widespread application of numerous therapeutic approaches focusing on the management of factors mentioned above [11–13]. Therefore, interventions that could effectively delay the progression of DN are greatly required.

In China, traditional Chinese medicine (TCM) has been widely used in the treatment of diabetes and its complications for a long time [14]; TCM has lots of advantages over the conventional medical approaches in the prevention of diabetic complications because of less toxicity and/or side effects [15–17]. In this review, we will explore the advance of herbal TCM treatment on DN in recent 10 years, based on the experimental and clinical studies to note the scientific basis for the therapeutic effects of TCM on DN.

## 2. Applications of TCM in DN

Plants have been widely used for medical purposes long before recorded history [18]. In China, TCM emerged and influenced the surrounding countries such as Japan and South Korea; increasing popularity of TCM caused great interests in laboratory and clinical investigations in lots of diseases on its efficiency and action mechanism. TCM manifests as herbal medicine, acupuncture, moxibustion, massage, dietary therapy, and physical exercise including shadow boxing and Qigong, and herbal remedies are the focus of TCM in mainland China [19] and acupuncture is prevalent in the United States [18]. Under the urgent need for the treatment of DN, we focus on the update of the efficient herbal TCM preparations, single herbal TCM, and/or monomers from herbal TCM in DN related clinical and experimental trials, through which we explore the effective herbal TCM for DN and clearly put forward underlying mechanism in the treatment of DN.

### 2.1. TCM Preparations in DN

TCM preparations are applied as decoction, pill, and capsule in the treatment of DN. We will introduce the TCM preparations in alphabetical order about components of TCM preparations, therapeutic effects in clinical or experimental studies, and relevant mechanism. All the mentioned TCM preparations in this review are listed in Table 1.

#### 2.1.1. Chaihuang Yishen Granule (CHYS)

Chaihuang Yishen granule (CHYS, also called Qilong-Lishui granule) is composed of radix astragali,* Dioscorea nipponica*, radix bupleuri,* Angelica sinensis*,* Pyrrosia petiolosa*,* Polyporus umbellatus*, and* Hirudo nipponica*. A recent study in STZ plus uninephrectomized induced rats showed that CHYS could be a therapeutic agent for DN by blocking TGF-*β*/Smad3-mediated renal fibrosis [20].

#### 2.1.2. Compound Rhizoma Coptidis Capsule (CRCC)

Compound rhizoma coptidis capsule (CRCC) is composed of rhizoma coptidis, Kudzu root, dwarf lilyturf,and Loquat leaf. CRCC has been shown to protect renal function and slow down the progression of DN by the suppression of TGF-*β*1 and type IV collagen expression in STZ induced diabetic rats [21].

#### 2.1.3. Compound Shenhua Tablet (CST)

Compound Shenhua Tablet (CST), is composed of radix astragali, fructus ligustri lucidi, rhizoma* zedoaria*, and honeysuckle. CST treatment in STZ induced diabetic rats showed that urine mAlb, Scr, BUN, Glu, TG, and TC were significantly lower than the diabetic model group [22].

#### 2.1.4. Danggui Buxue Tang (DBT)

Danggui buxue tang (DBT), a preparation including radix astragaliandradix* Angelica sinensis*, has been shown to partially attenuate the increases in blood glucose, TG, and CHO, and DBT was supposed to retard DN progression by suppressing TGF-*β*1 expression in STZ induced diabetic rats [23]. In the HG stimulated glomerular mesangial cells, DBT could inhibit cell proliferation and expression of LN, FN, and collagen IV indicating the renoprotective effect of DBT on DN at the early stages [24].

#### 2.1.5. Danggui Shaoyao San (DSS)

Danggui Shaoyao San (DSS) is a famous TCM formula comprising six herbal medicines: radix Paeoniae Alba, radix* Angelica sinensis*, rhizoma Chuanxiong,* Poria cocos*, rhizoma* Atractylodis macrocephala*, and rhizoma Alismatis. DSS has been shown to protect renal function in STZ induced diabetic rats through regulating plasma glucose and attenuating AGEs expression in diabetic glomeruli [25].

#### 2.1.6. Fufang Xue Shuan Tong (FXST)

Fufang Xue Shuan Tong (FXST) capsule is composed of radix notoginseng,* Salvia miltiorrhiza*, XuanShen, and radix astragali and has been used to treat DN for many years. High dose of FXST treatment could prevent glomerular hypertrophy and mesangial matrix expansion through regulation of oxidative stress including increasing SOD activities and decreasing MDA levels in the kidney of HFD-fed plus STZ induced rats [26].

#### 2.1.7. Hachimijiogan (HJG)

A most popular herbal medicine in Japanese Kampo, Hachimijiogan (HJG, Ba Wei Di Huang Wan in Chinese), is extracted from a mixture of* Rehmannia* radix, corni fructus,* Dioscorea* rhizome, Hoelen, Alismatis rhizome, Moutan cortex, Cinnamomi cortex, and Aconiti tuber. In subtotal nephrectomy plus STZ induced rats, HJG could reduce blood glucose and urinary protein excretion levels and increase Ccr; furthermore, HJG could ameliorate oxidative stress and AGEs formation associated with DN and subsequently prevent the development of renal lesions including glomerular sclerosis, tubulointerstitial lesions, mesangial expansions, and atherosclerosis [27]. In spontaneous diabetic WBN/Kob rats with DN, HJG could prevent DN progression through several established biomarkers in plasma [28] and by reducing renal oxidative injury and expression of FN and TGF-*β*1 proteins [29]. In OLETF rats, HJG could reduce TGF-*β*1, FN, iNOS, and COX-2 expressions in kidney cortex, urinary protein excretion was decreased, Ccr levels were improved, and serum glycosylated protein and AGEs were reduced effectively; data mentioned above suggested that HJG has beneficial effect on the DN progression [30].

#### 2.1.8. Hu-Lu-Ba-Wan (HLBW)

Hu-Lu-Ba-Wan (HLBW), composed of* Trigonella foenum-graecum* L. (*TFG*) and* Psoralea corylifolia* L. (*PC*), has been shown to improve hyperglycemia, hyperlipidemia, and proteinuria in the HFD-fed plus STZ induced rats and could play renoprotective effect in attenuating renal oxidative stress via PKC-*α*/NADPH oxidative pathway [31].

#### 2.1.9. Liuwei Dihuang Pill (LDP)

Liuwei Dihuang Pill (LDP), one formulation in the ancient Chinese medicine, includes six crude drugs:* Rehmannia glutinosa*, fructus corni, cortex Mountain,* Dioscorea opposita*,* Poria cocos*, and* Alisma*. A previous study in DN patients showed that LDP could improve symptoms and signs of DN and inhibit erythrocyte aldose reductase (EAR) activity and lower UAER levels, *β*_2_-microglobulin in blood and urine without affecting blood glucose, lipids, and mean arterial pressure [32]. LDP treatment in type 2 diabetic patients was found to be associated with the relief of DN [33]. Liuwei Dihuang (LW) decoction has also been proven to relieve early DN abnormalities mediated by suppression of renal entothelin-1-reactive oxidative species (ET-ROS) system and escalating MMPs activity [57], and LW without fructus corni could alleviate DN by combined suppression of ET-ROS axis with modulating hypoglycemic effects in STZ induced diabetic rats [58].

#### 2.1.10. Oryeongsan (Wulingsan)

Oryeongsan (Wulingsan), also named as Hoelen Five Herb Formula, is composed of five crude drugs:* Poria*, Alismatis rhizoma,* Polyporus umbellatus (Pers.) Fries*, rhizoma* Atractylodis macrocephala*, and Ramulus Cinnamomi Cassiae. A previous study showed that Oryeongsan could play renal protective roles in lowering plasma glucose and ameliorating glycation-mediated renal damage through attenuating increased NF-*κ*B and TGF-*β*1 expression in STZ induced diabetic rats [34]. Further study showed that Oryeongsan could ameliorate insulin resistance and DN in db/db mice by disturbing the TGF-*β*1/Smads pathway [35].

#### 2.1.11. Qizhi Jiangtang Capsule (QJC)

Qizhi Jiangtang Capsule (QJC) is composed of four crude drugs: radix astragali,* Hirudo*,* Rehmannia* root, and rhizoma Polygonati. In a multicenter randomized clinical study, QJC has been shown to reduce urinary protein effectively and delay the progression of renal function in treating 3b DN patients [36].

#### 2.1.12. Qiwei Granule (QWG)

Qiwei Granule (QWG) is composed of radix astragali, radix* Rehmannia*,* Euonymus alatus*, and Rhubarb. QWG could alleviate renal pathological changes and decrease TGF-*β*1 expression in the type 2 diabetic KK-Ay mice, which suggested that QWG could play roles in preventing and curing DN [37].

#### 2.1.13. Shenkangwan (SKW)

Shenkangwan (SKW) is composed of two crude drugs: radix astragaliand Herba Leonuri. SKW was reported to protect renal function by increasing NO production and decreasing TGF-*β*1 excretion in the mesangial cells from diabetic rats [38]; in diabetic rats SKW could reduce FN expression in kidney [59] while in rat mesangial cells SKW has been shown to suppress FN secretion via TGF-*β*1 signal way [60]. Another study showed that in STZ induced diabetic rats SKW could protect renal function and alleviate the functional and structural damage of podocytes possibly by reducing desmin and increasing podocin expression [61], and SKW could offer renal protection against DN by reducing Ang II levels in the plasma and kidney tissues and inhibiting renal AT(1)R expressions [39]. All the data supply precise mechanism of SKW treating DN.

#### 2.1.14. Supplementing Qi and Activating Blood Circulation (SQABC)

Supplementing Qi and activating blood circulation (SQABC) is composed of radix astragali and* Salvia miltiorrhiza* and has been shown to reduce 24 h urinary protein excretion and improve tubular reabsorption function by enhancing renal tissue activity of antioxidant and upregulating megalin expression in tubular epithelial cells in STZ induced diabetic rats [40]. Another* in vitro* study showed that supplementing Qi and activating blood circulation could protect HG injured NRK-52E cells and improve protein uptake by increasing megalin expression [41].

#### 2.1.15. Tangshen Formula (TSF)

Tangshen Formula (TSF) is composed of* Astragalus*, raw* Rehmannia* root, sanchi root,* Euonymus* branchlet, rhubarb, bitter orange, and dogwood fruit. TSF has been shown to regulate and improve phospholipids metabolism in DN patients related with inhibition of PKC pathway and the corresponding reduction of phospholipase A2 activity [42]. In a study on the Hcy metabolism of DN patients, TSF could improve* in vivo* hypomethylation and oxidative stress showing similar favorable effect to western medicine in the treatment of DN [43]. In the molecular mechanism study using a db/db mice model, TSF showed beneficial effects on DN treatment via regulating the JAK/STAT/SOCS signaling pathway [44].

#### 2.1.16. Tongshenluo (TSL) Capsule

Tongshenluo (TSL) capsule is composed of six crude drugs: radix astragali, radix* Rehmannia*, leech, bile south star,* Artemisia anomala*, and Ze lan. TSL has been shown to decrease the levels of FBG, HbA1c, and urinary mAlb in the subtotal nephrectomy plus STZ induced diabetic rats and decrease the components of ECM through downregulating TGF-*β*1 and TIMP-2 and upregulating MMP-2 expression [45].

#### 2.1.17. Tongxinluo (TXL)

Tongxinluo (TXL) capsule include 8 crude drugs: scorpion, leech,* centipede*,* ground beetle*,* cicada*,* borneol*, radix paeoniae rubra, and ginseng. TXL capsule has been shown to improve renal function, repair the renal tubular interstitial damage, and delay the progression of DN patients by reducing plasma ET-1 and UAER [46]. TXL was also demonstrated to ameliorate renal function and structure by regulating miRNA-21-induced EMT, suggesting miRNA-21 may be one of the therapeutic targets for TXLC in DN [47]. Another study showed that TXL could also successfully inhibit TGF-*β*1 induced EMT in DN [62].

#### 2.1.18. Xiao Chai Hu Tang (XCHT)

Xiao Chai Hu Tang (XCHT, Shosaiko-to in Japanese) is a herbal drug formula extensively applied in TCM and Japanese Kampo medicine, comprising seven medicinal plants: radix bupleuri,* Scutellaria baicalensis* Georgi radix,* Panax ginseng*,* Pinellia ternata* tuber,* Glycyrrhiza glabra*, ginger slice, and* Zizyphus vulgaris* Lam.* fructus*. XCHT has been shown to decrease the expression of TGF-*β*1, FN, and collagen IV accompanied with increased BMP-7 expression in STZ induced diabetic mice and HG stimulated RMC, which was mediated through decreasing oxidative stress and productions of TGF-*β*1, FN, and collagen IV in renal cortex during the development of DN [48].

#### 2.1.19. Xiaoke Granule (XKG)

Xiaoke granule (XKG, Xiaoke Keli in Chinese) includes four crude drugs: radix astragali, Mountain* Cornus*, leech, and winged euonymus twig. It was reported that XKG could decrease fasting blood pressure and 24 h urinary protein excretion in the 3/4 nephrectomy and STZ induced diabetic rats groups [49]. In the subsequent mechanism study, XKG was proved to exert renal protective effect in DN through downregulating TGF-*β*1 expression in rat mesangial cells [50].

#### 2.1.20. Xiexin Decoction (XXD)

Xiexin decoction (XXD) is composed of three crude drugs including radix et rhizoma rhei, rhizoma coptidis, and radix* Scutellaria* and has been used for the treatment of DM for at least 1700 years. One study in HFD-fed plus STZ induced rats showed that XXD could attenuate albuminuria and renal pathological changes, reduce AGEs, inhibit RAGE and inflammation factors expression, suppress NF-*κ*B, and downregulate renal TGF-*β*1. All these data suggested that renal protective potential of XXD was involved in inhibition of inflammation through downregulating NF-*κ*B pathway, reducing renal AGEs and RAGE in diabetic rats [51]. A recent study of XXD components in db/db mice showed that multicomponent herbal therapeutic formulations could be a useful approach for the treatment of DN through reducing the expression of NF-*κ*B and TGF-*β*1 [52].

#### 2.1.21. Xianzhen Tablet (XZT)

Xianzhen tablet (XZT, a Chinese patent compound recipe), is composed of astragali radix, radix* Rehmannia*, fructus ligustri lucidi,* Scutellaria baicalensis* Georgi, rhizoma coptidis, dodder weed, fairy spleen, and* Salvia miltiorrhiza*. XZT was reported to decrease blood glucose and HbA1c in diabetic rats, improve renal function, ameliorate proteinuria, and reduce glomerular extracellular matrix expansion and thickness of basement membrane, which was mediated by the inhibition of AGEs accumulation and RAGE mRNA levels in the kidney cortex of STZ induced diabetic rats [53].

#### 2.1.22. Zhibai Dihuang Pill (ZDP)

Zhibai Dihuang Pill (ZDP) is one of the TCM preparations, composed of rhizoma anemarrhenae, cortex phellodendri, radix* Rehmannia* preparata, rhizoma* Dioscorea*, fructus corni, cortex Moutan, rhizoma Alismatis, and* Poria*. ZDP has been revealed to have protective effects in experimental DN animal models and DN patients. In a recent metabonomic analysis of ZDP in the treatment of STZ induced diabetic rats, ZDP could ameliorate DN by intervening in some dominant metabolic pathways such as inhibiting glucose and lipid metabolism and enhancing methylamine metabolism [54].

#### 2.1.23. Zao Huang Mixture (ZHM)

Zao Huang Mixture (ZHM) is composed of extracts of* Sargassum* and rhizoma rhei. One study has shown that ZHM could prevent the process of DN by decreasing the expression of TGF-*β*1 and type IV collagen in HG stimulated human glomerular mesangial cells [55].

#### 2.1.24. Zhenqing Recipe (ZQR)

Zhenqing Recipe (ZQR), a Chinese herbal prescription composed of 3 crude drugs: fructus ligustri lucidi,* Eclipta prostrate*, and* Dioscorea opposite*, has been used to improve renal function of DN patients. In the study for the underlying mechanism, ZQR has been shown to inhibit the overexpression of TGF-*β*1 and FN in the renal cortex of HFD-fed plus STZ induced diabetic rats; its renoprotective effect was mediated by inhibiting SREBP-1c overexpression and its target genes including ACC and FAS [17].

#### 2.1.25. Zishentongluo (ZSTL)

Zishentongluo (ZSTL) is composed of eleven Chinese herbs: raw* Astragalus*,* Angelica*, safflower, zedoary turmeric, Dodder, radix* Rehmannia*, dogwood,* Poria*,* Epimedium*, earthworm, and* Schisandra*. ZSTL has been shown to be superior to benazepril in improving the metabolic and renal function in DN patients at early stage partially by modifying ANP, VEGF, and ET-1 expressions [56].

### 2.2. Monomers/Single TCM in DN

With the development of modernization of TCM preparations and applications in the treatment of DN, pharmacological complexity is difficult to be distinguished for the precise underlying mechanism, and, to avoid the toxicity and side effects, there is an increasing interest of single herbal TCM and/or monomers from herbal TCM in the treatment of DN, and they are more appropriate than TCM preparations to clarify the precise action mechanism on DN. All the single herbal TCM and monomers are listed in Table 2.

#### 2.2.1. *Astragalus*/Radix Astragali

*Astragalus* (Huang Qi in Chinese), also named as radix astragali, is a TCM from Mongolian milkvetch or* membranaceus* milkvetch. A meta-analysis comprising 25 studies showed that* Astragalus* injection had more therapeutic effect in DN patients such as decreasing BUN, Scr, and urine protein and improving Ccr and serum albumin level [63], and rebalancing TGF-*β*/Smad signaling could be a potential mechanism to prevent DN in KK-Ay mice [64].* Astragalus* may protect diabetic rats kidney mediated by downregulation of Tie-2 [110], and radix astragali was reported to upregulate c-met expression in human kidney fibroblasts to delay the progression of DN [65]. Two major isoflavonoids in radix astragali, calycosin and calycosin-7-O-beta-D-glucoside, could inhibit HG induced mesangial cell early proliferation and AGEs-mediated cell apoptosis, suggesting these two isoflavonoids have therapeutic potential to prevent the progression of DN [111]. A recent review showed that total polysaccharides, flavonoids fractions, saponins, and several isolated compounds have antidiabetic potentials, which throw light upon further investigations that should be conducted on the treatment of DN and relevant underlying mechanism [112]. Astragaloside IV (ASI) in radix astragali is considered to be an active constituents; ASI could inhibit human tubular epithelial cells apoptosis and reduce TGF-*β*1 expression, suggesting a new treatment for DN probably mediated by the inhibition of p38 MAPK pathway activation and HGF overproduction [113].

#### 2.2.2. Berberine (BBR)

Berberine (BBR), an effective compound of herbal TCM, includes* Coptis chinensis*,* Hydrastis Canadensis*,* Berberis aristata*,* Berberis aquifolium*, and* Arcangelisia flava*.

BBR treatment could restore renal functional parameters, improve glucose and lipid metabolism disorders, suppress alterations of histological and ultrastructural changes in kidney, and increase cAMP levels in HFD-fed plus STZ induced diabetic rats, and the renal protective effect is exerted by modulating the G protein-coupled receptor kinases (GRKs) in G protein-AC-cAMP signaling pathway [66]. A previous study showed that BBR-containing TCM could increase glucose uptake and lipid oxidation with insulin sensitivity in Zucker diabetic fatty rats [16].

#### 2.2.3. CLL/Curcumin

*Curcuma longa* L. (CLL) has been widely used to prevent diabetic vascular complications in recent years. Curcumin and demethoxycurcumin are isolated from CLL and have been shown to potentially protect DN by reducing AGE-induced oxidative stress and restoring AGE-induced mesangial cell apoptosis [68]. In the treatment of DN in db/db mice, curcumin has been shown to decrease albuminuria and attenuate glomerular sclerosis by inhibiting phosphorylation of STAT3 and degradation of I*κ*B [67]. A systemic review and meta-analysis of fourteen randomized controlled trials suggested that curcumin has protective potentials on the kidneys of diabetic rats/mice [114].

#### 2.2.4. 2-Dodecyl-6-methoxycyclohexa-2,5-diene-1,4-dione (DMDD)

2-Dodecyl-6-methoxycyclohexa-2,5-diene-1,4-dione (DMDD), isolated from the tuberous roots of* A. carambola* L. (Oxalidaceae), has been shown to enhance the reduced SOD activities in the kidney of KK-Ay mice and inhibit the progression of DN through decreasing AGEs and TGF-*β*1 levels [69].

#### 2.2.5. Dracorhodin Perchlorate (DP)

Dracorhodin perchlorate (DP), one of the main compositions of Dragon's blood, has been shown to prevent and retard renal fibrosis of DN partially through inhibiting SGK1 and FN expression in human mesangial cells [70].

#### 2.2.6. EGB

*Ginkgo biloba* extract (EGB), taken from the leaves of* Ginkgo biloba*, is a mixture containing flavonoid glycosides and has been proven to ameliorate hemodynamics, suppress PAF and ACE activities, scavenge ROS, relax vascular smooth muscles, and suppress AGEs expression. In a previous study on DN patients, EGB treatment has been shown to decrease urinary mALB, *α*1-MG, IgG, TF, RBP, and NAG in DN patients compared with control group, which suggested that EGB has renoprotective effect on the early DN [71]. The subsequent mechanism study showed that EGB could suppress rat mesangial cells hypertrophy and ECM accumulation through decreasing Smad2/3 and TGF-*β*1 and increasing Smad7 [73], while in DN patients EGB has been proven to retard early DN development through decreasing serum sICAM-1 and sVCAM-1 levels [72].

#### 2.2.7. Flos* Abelmoschus manihot*

Flos* Abelmoschus manihot* (Huangshukuihua in Chinese) has been widely used as the neuroprotective drug for cerebral ischemic reperfusion injury. Total flavone glycosides of flos* A. manihot* (TFA) contain 7 identified flavone glycosides. TFA pretreatment has been shown to prevent renal damage and podocyte apoptosis in STZ induced rats [76]. A meta-analysis of 27 randomized controlled trials showed that flos* Abelmoschus manihot* had significant effect on renal function in the treatment of DN deserving further investigation [115].

#### 2.2.8. Genipin

Genipin is a glycone derived from geniposide present in fruit of* Gardenia jasminoides*. Genipin has been proven to ameliorate body weight loss and urine albumin leakage, attenuate GBM thickness, and restore the podocyte expression of podocin and WT1 in diabetic mice; the protective effect of Genipin on DN is probably through suppressing the upregulation of mitochondrial UCP2 in STZ induced diabetic mice kidneys [77].

#### 2.2.9. *Houttuynia cordata* Thunb. (HCT)

*Houttuynia cordata* Thunb. (HCT, Yu Xing Cao in Chinese), pungent in taste and cool in nature, has been reported to reduce urinary proteins in the patients with nephrotic syndrome; HCT has also been shown to protect diabetic kidney function through decreasing the expression of TGF-*β*1 and increasing the expression of BMP-7 [78].

#### 2.2.10. Icariin

Icariin is a major constituent of flavonoid extracted from the plant herba epimedii and has been shown to relieve renal damage in STZ induced diabetic rats through inhibiting the expression of TGF-*β*1 and collagen IV protein [79].

#### 2.2.11. LAB

Lithospermate B (LAB), a tetramer of caffeic acid isolated from* Salvia miltiorrhiza* radix, was identified as antioxidant and PKC inhibitor in the renoprotective effects under diabetic conditions* in vivo* and* in vitro* [80]. In the STZ induced diabetic rats, delayed LAB treatment could inhibit renal MDA, microalbuminuria, mesangial expansion, and glomerular hypertrophy, and in mesangial cells LAB could inhibit HG and H_2_O_2_ induced TGF-*β*1 and FN secretion, HG induced intracellular PKC activation, and ROS generation, which suggested that LAB could significantly suppress the progression of diabetic renal injury. A recent study showed that LAB could prevent diabetic atherosclerosis by induction of the Nrf2-ARE-NQO1 pathway to inhibit VSMCs proliferation and migration and vascular damage [81]. All these findings suggested that LAB could be a new therapeutic agent in the treatment of DN. In the subsequent study,* Salvia miltiorrhiza* could protect STZ induced diabetic rats by inhibiting the overexpression of TGF-*β*1, CTGF, PAI-1, and FN in renal cortex.

#### 2.2.12. LBP

*Lycium barbarum* polysaccharide (LBP) is extracted from the fruit of goji berry(Solanaceae); LBP4 has been shown to protect STZ induced diabetic kidney function via decreasing the activation of ERK1/2 through the involvement of PKC in mesangial cells [82].

#### 2.2.13. LGP

Lyoniresinol 3 alpha-O-beta-D-glucopyranoside (LGP) is isolated from* Averrhoa carambola* L. (Oxalidaceae) root (ACLR), including two chiral lignin glucosides: LGP1 and LGP2. LGP1 treatment has been shown to decrease hyperglycemia and the expression of related proteins including NF-*κ*B, caspase-3, caspase-8, caspase-9, and Bax in STZ induced diabetic mice. LGP1 also could alleviate glomerular hypertrophy, excessive ECM accumulation, and glomerular and tubular basement membrane thickness. All these data suggested that LGP1 could be a potential therapeutic agent in DN [83].

#### 2.2.14. Ligustrazine

Ligustrazine, a bioactive component of Chuangxiong, has been widely used in the treatment of vascular diseases such as myocardial and cerebral infarction in China. A meta-analysis of 25 studies showed that Ligustrazine has therapeutic effect to improve renal function and reduce urine protein excretion in DN patients [84]. Further studies should be conducted to reveal the underlying mechanism for the treatment on DN.

#### 2.2.15. MC

Moutan cortex (MC), the root bark of* Paeonia suffruticosa*, has been shown to have the protective effect against atherosclerosis and inflammation and inhibitory effect on the production of ROS. MC was reported to increase activity of SOD, GSH-PX, and CAT and reduce MDA* in vitro* or* in vivo*; furthermore, MC could decrease blood glucose, Scr, and urine protein in HFD-fed plus STZ induced diabetic rats, which suggested that MC has renal protective effect in AGEs-induced mesangial cell dysfunction through attenuating oxidative stress pathway [85], while, in AGEs-induced rat mesangial cells, MC could inhibit FN and collagen IV expression in matrix [86]. Apart from the abovementioned evidence of renal protective effect on DN, MC could ameliorate activity on the inflammation via target of RAGE* in vitro* or* in vivo* [87].

#### 2.2.16. Morroniside

Corni fructus, a constituent of HJG, used as a traditional medicine in China and Japan, has been shown to be superior to aminoguanidine treatment in suppressing hyperglycemia, proteinuria, renal AGE formation, and TGF-*β*1 expression in STZ induced diabetic rats [116]. Morroniside, isolated from corni fructus, could exhibit protective effects against STZ induced renal damage by inhibiting hyperglycemia and oxidative stress [88]. Another study showed that components of corni fructus could play protective effect on early stage of DN in type 2 diabetic rats mediated by the regulation of podocytes. Loganin from corni fructus and its derivatives could inhibit the expression of FN and IL-6 in the HG stimulated mesangial cells, which supported the traditional use of corni fructus in DN and relevant kidney diseases [117].

#### 2.2.17. Panax Notoginoside (PNS)

Panax notoginoside (PNS) is extracted from radix notoginseng and has been shown to protect kidney in type 1 diabetic rats at early stage through inhibiting the expression of VEGF protein and enhancing BMP-7 expression in the kidney [89]. Another report showed that the protective effect of PNS in kidney was mediated by inhibiting TGF-*β*1 expression and enhancing the expression of Smad7 [90]. Ginsenoside Rg1, an active ingredient isolated from PNS, has been shown to improve the renal pathological changes in STZ induced diabetic rats through reducing TGF-*β*1 expression and inflammatory reaction factors including CRP and TNF-*α* [118]. Ginsenoside Rg1 also could effectively relieve aldosterone-induced oxidative stress through which it indirectly inhibits aldosterone-induced podocyte autophagy [119].

#### 2.2.18. Puerarin

Puerarin, 7-hydroxy-3-(4-hydroxyphenyl)-1-benzopyran-4-one-8-b-D-glucopyranosid-e, is one of the major isoflavonoid compounds from the root of* Pueraria candollei* wall of Leguminosae family. A previous study showed that Puerarin could protect DN rats by inhibiting collagen IV expression [91]; further study in STZ induced diabetic rats showed that Puerarin could protect kidney function through downregulating MMP-9 and attenuating eNOS expression [92, 93].

#### 2.2.19. *Rehmannia* Radix (Di Huang)

*Rehmannia* radix (Di Huang) was mostly mentioned and investigated; it has been proven to reduce hyperglycemia, ameliorate renal dysfunction, prevent senility, and improve hemorheology. In a previous experimental study* Rehmannia* radix has been shown to inhibit the progression of DN [120]. Catalpol is an iridoid glucoside compound mainly present in* Rehmannia* radix and other plants amd has been shown to reduce ECM accumulation by inhibiting the expression of TGF-*β*1, CTGF, and Ang II in HFD-fed plus STZ induced diabetic rats [121].

#### 2.2.20. Rhein

Rhein (4,5-dihydroxyanthraquinone-2-carboxylic acid) is purified from rhubarb (*Rheum officinale*). Rhein has shown reduction of UAE faster than simvastatin and decrease of ECM levels along with decreased TGF-*β*1 and FN immunohistochemistry expression in db/db renal tissue, which was supposed via regulation of dyslipidemia [94]. Another study showed that Rhein could inhibit the hypertrophy of rat renal proximal tubular epithelial cells stimulated by HG and Ang II [95].

#### 2.2.21. *Rhodiola rosea*

*Rhodiola rosea* (*R. rosea*) is grown at northern latitudes and high altitudes of the world;* Rhodiola rosea* extract has been used to protect kidney function including reducing FBG, TC, TG, Ccr, and 24 h urinary albumin in HFD-fed plus STZ induced diabetic rats through decreasing renal expression of TGF-*β*1 [96].

#### 2.2.22. *Rosa laevigata* Michx. (RLM)

*Rosa laevigata* Michx. (RLM), a commonly used TCM for the treatment of urinary tract infection and antioxidative treatment, could play a critical role in the pathogenesis of DN through increasing the activity of SOD and total antioxidant capacity, decreasing MDA and ROS levels, and inhibiting NF-*κ*B p65 and MCP-1 expression following increased I*κ*B protein expression in STZ induced diabetic rats; all the data suggested that RLM could be a therapeutic potential for DN [97].

#### 2.2.23. Sequoyitol

Sequoyitol is a natural compound present in a lot of plants (e.g.,* Aristolochia arcuata*,* Amentotaxus yunnanensis*, and* Crossostephium chinensis*); oral and subcutaneous administrations of sequoyitol could ameliorate hyperglycemia and glucose intolerance in ob/ob mice. Sequoyitol has been shown to ameliorate the progression of DN in HFD-fed plus STZ induced rats through glucose-lowering effects, antioxidant activity, and regulation of TGF-*β*1 expression [98].

#### 2.2.24. SF/FA

Sodium ferulate (SF), extracted from* Angelica sinensis*,* Lignsticum chuangxiong*,* Cimicifuga heracleifolia*, and other plants, has platelet aggregation inhibitory, antithrombotic, and antioxidant activities in animals and humans. A preliminary study on DN patients showed that SF could lower UAER level and improve renal function through decreasing endothelin (ET) and inhibiting the combination of ET with its receptor [99]. A meta-analysis of 14 randomized controlled trials involving 906 patients showed that SF is superior in reducing UAER, ET, BUN, Scr, and TC and increasing HDL-c without affecting FBG and TG [122]. Ferulic acid (FA) is a phenolic acid extracted from the seeds and leaves of most plants and has antioxidant activities, hypoglycemic and hypolipidemic effects, hypotensive effects, and anti-inflammatory effects. In the FA treated OLETF rats, blood glucose and urinary ACR were decreased significantly; in renal histopathology glomerular basement membrane thickness and mesangial matrix expansion were decreased through reducing oxidative stress and inflammation [74, 75].

#### 2.2.25. Skimmin

Skimmin, a major active component from* Hydrangea paniculata*, has been reported to decrease Scr and blood glucose level and alleviate glomerular segmental sclerosis and incidence of tubular vacuolar degeneration by downregulating the TGF-*β*1 and TGF-*β*1 receptor I expression in STZ induced diabetic rats [100].

#### 2.2.26. SM

*Salvia miltiorrhiza* (SM, commonly known as Danshen in Chinese) has been shown to have the anti-inflammatory, antioxidative, and organ protective effects. A previous study showed that SM could protect STZ induced diabetic rats from DN by suppressing the overexpression of TGF-*β*1, CTGF, PAI-1, and FN in renal cortex [101]. Another study showed that SM could ameliorate TGF-*β*1 levels in serum and kidney and reduce the levels of collagen IV ED-1 and RAGE in the diabetic kidney [102]. Danshen injection, the aqueous extracts of SM, could protect diabetic rats associated with preservation of tubular function and structure from hyperglycemia induced oxidative stress, advanced glycation stress, and megalin expression deletion [103].

#### 2.2.27. TGP

Total glucosides of paeony (TGP), extracted from the root of* Paeonia lactiflora* Pall., have been shown to have the therapeutic effect in the experimental DN. TGP treatment in the STZ induced diabetic rats could prevent diabetic renal damage against oxidative stress through decreasing upregulated p-p38 MAPK and NF-*κ*B P65 expressions [104]. And, in the HFD-fed plus STZ induced rats, TGP could improve kidney damage and delay the development of DN by inhibiting Wnt/beta-catenin signaling pathway [105].

#### 2.2.28. TMP

Tetramethylpyrazine (TMP) is isolated from* Ligusticum chuanxiong* and has been used in the treatment of stroke and cardiovascular diseases. TMP was reported to reduce diabetic kidney damage partially by downregulating the expression of VEGF in the kidney [106].

#### 2.2.29. Triptolide/GTW/TwHF

Triptolide, active diterpene purified from* Tripterygium wilfordii* Hook. F. (TwHF), has been reported to have anti-inflammatory, antioxidative, immunosuppressive, and podocyte-protective effects. A recent study showed that triptolide could attenuate albuminuria in db/db diabetic mice accompanied with alleviated glomerular hypertrophy and podocyte injury, while inflammation and dyslipidemia were also attenuated [107]. Triptolide is one of the major active components of multiglycoside of TwHF (GTW), and GTW has been applied extensively for the treatment of CKD in China as an anti-inflammatory agent. GTW could prevent glomerular lesion in STZ induced diabetic model through decreasing urine albumin and ameliorating glomerular sclerosis [123]. A recent study showed that TwHF could prevent podocyte injury of DN patients, which may be partly mediated by downregulating the expression of OPN, CTGF, and TGF-*β*1 [108].

#### 2.2.30. Volatile Oil of* Magnolia biondii* Pamp. (VOMBP)

Volatile oil of* Magnolia biondii* Pamp. (VOMBP), extracted from herbal TCM* Magnolia biondii* Pamp., has been reported to protect the kidney in STZ induced diabetic rats by inhibiting the expression of P-selectin in serum and renal tissue [109].

### 2.3. TCMs Combined Therapy with Western Medicines in DN

Apart from the TCM preparations and single TCM applications in DN, TCMs combined with western medicines have been indicated. Mostly used western medicines were ACEI/ARBs, and combination styles included Tangshenling (TSL) with telmisartan [124] in diabetic patients or TSL with benazepril in STZ induced rats [125], triptolide with benazepril in DN patients [126], Bailing Capsule (BC) and benazepril in DN patients [127], and safflower yellow powder injection with benazepril in DN patients [128]. Another report is about Tangshenqing (TSQ) combined with alprostadil in the treatment of DN patients [129]. All data suggested that effects of TCMs combined therapy with western medicines were superior to western medicines treatment alone.

## 3. Conclusions and Perspectives

Although there are almost no side effects mentioned in numerous scientific reports, a lot of scientific researches indicate that herbal TCM preparations have renal protective effects on DN according to respective factors, complexity, and variability of TCM preparations still presenting challenges for clinicians seeking scientific evidence to support TCM application in drug discovery. In order to avoid the toxicity and side effects of TCM formulas, there is increasing interest in studying single herbal TCM especially monomers from single herbal TCM on DN. In this review, we found that monomers such as Berberine, curcumin, Ginsenoside Rg1, Puerain, Rhein, and Ferulic acid have specific protective effect on DN. To translate the therapeutic potentials for DN into reality, placebo-controlled and randomized controlled clinical trials of single herbal TCM and/or monomers from herbal TCM are essential in the future, and prompt meta-analysis is an effective alternative.

## Figures and Tables

**Table 1:** Applications of herbal TCM Preparations in DN.

Name	Origins	Methods	Results	Pathways
CHYS	Radix astragali*, Dioscorea nipponica, *radix bupleuri*, Angelica sinensis, Pyrrosia petiolosa, Polyporus umbellatus*, and* Hirudo nipponica *	*Type 1 diabetic animal study* (STZ + nephrectomized rat)	Inhibiting 24 h proteinuria and progressive renal fibrosis (glomerulosclerosis index, tubulointerstitial fibrosis index, and upregulation of ECM), upregulating Smad7, and downregulating TGF-*β*1, TGF-*β*R, Smad3 activation, and miRNA-21	[20]

CRCC	Rhizoma coptidis, Kudzu root, dwarf lilyturf, and loquat leaf	*Type 1 diabetic animal study* (STZ induced rats)	Reducing FBG, BUN, Cr, Upro levels and TGF-*β*1, and collagen IV expressions and alleviating pathological lesions of kidney	Through TGF-*β*1 pathway [21]

CST	Radix astragali, fructus ligustri lucidi,Rhizoma *zedoaria*, and honeysuckle	*Type 1 diabetic animal study* (STZ induced rats)	Decreasing urine mAlb, Scr, BUN, Glu, TG, and TC	[22]

DBT	*Angelica sinensis and Astragalus membranaceus *	*Type 1 diabetic animal study* (STZ induced rats)	Attenuating the increases in blood glucose, TG and CHO, and TGF-*β*1 expression in kidney	Through TGF-*β*1 way [23, 24]
		*Cellular study* (mesangial cells)	Inhibit cell proliferation and expression of LN, FN, and collagen IV	

DSS	Radix Paeoniae Alba, radix* Angelica sinensis, *rhizoma* Chuanxiong*,* Poria cocos*, rhizoma* Atractylodis macrocephala*,and Alismatis rhizome	*Type 1 diabetic animal study* (STZ induced rats)	Decreasing FBG and attenuating AGEs expression in diabetic glomeruli	Through modulating oxidative stress via AGEs expression [25]

FXST	SanQi, DanShen, XuanShen, and HuangQi	*Type 2 diabetic animal study* (HFD + STZ induced rats)	Preventing glomerular hypertrophy and mesangial matrix expansion	Through regulating oxidative stress [26]

HJG	*Rehmanniae *radix*, Corni fructus, Dioscorea *rhizome, Hoelen, Alismatis rhizome, Moutan cortex, Cinnamomi cortex, and Aconiti tuber	*Type 1 diabetic animal study* (STZ + nephrectomized rat)	Reducing blood glucose and urinary protein excretion and increasing creatinine clearance, ameliorating oxidative stress and AGEs formation associated with DN, and preventing the development of renal lesions including glomerular sclerosis, tubulointerstitial lesions, mesangial expansions, and atherosclerosis	Inhibiting AGEs formation and sorbitol levels in kidney [27]
		*Type 1 diabetic animal study* (WBN/Kob rats)	Preventing diabetic kidney damage	Reducing renal oxidative injury and expression of FN/TGF-*β*1 proteins [28, 29]

HLBW	*Trigonella foenum-graecum *L. (*TFG*) and* Psoralea corylifolia *L. (*PC*)	*Type 2 diabetic animal study* (HFD + STZ induced rats)	Improving hyperglycemia, hyperlipidemia, and proteinuria	Through attenuating renal oxidative stress via PKC-*α*/NADPH oxidative pathway [31]

LDP	*Rehmannia glutinosa, *Cornel (manufactured), Moutancortex*, Yam*,* Poria cocos*, and *Alisma *	*Human study* (DN patients)	Improving symptoms and signs of DN, inhibiting EAR activity, lowering UAER levels, *β*_2_-microglobulin in blood, and urine, and relieving DN	[32, 33]

Oryeongsan	*Poria, Alismatis rhizoma, Polyporus umbellatus (Pers.) Fries,* rhizoma *Atractylodis macrocephala*, and Ramulus Cinnamomi Cassiae	*Type 1 diabetic animal study* (STZ induced rats)	Decreasing plasma glucose, UAER, and Ccr, attenuating mesangial matrix expansion, and downregulating increased NF-*κ*B, TGF-*β*1 expression, elevated AGEs, and FN accumulation	Through attenuating increased NF-*κ*B and TGF-*β*1 expression [34]
		*Type 2 diabetic animal study* (db/db mice)	Decreasing TC and TG, improving blood glucose, insulin, glucose tolerance, and HOMA-IR, Ccr, urine albumin, and BUN, and reducing TGF-*β*1, Smad2/4, collagen IV, CTGF, and TIMP	Through disturbing the TGF-*β*1/Smads pathway [35]

QJC	Radix astragali*, Hirudo, Rehmannia *root, and rhizoma Polygonati	*Human study* (DN patients)	Decreasing SBP and DBS, increasing ALB, and slowing down the increase of Scr and decrease of eGFR	[36]

QWG	Radix astragali, radix* Rehmanniae, Euonymus alatus*, and Rhubarb	*Type 2 diabetic animal study* (KK-Ay mice)	Alleviate renal pathological changes and decreasing TGF-*β*1 expression	Through inhibiting TGF-*β*1 expression [37]

SKW	Radix astragali, Herba Leonuri	*Type 1 diabetic animal study* (STZ induced rats)	Protecting renal function	Through increasing NO and decreasing TGF-*β*1 excretion; affecting podocytes special proteins expression [38]
		*Type 1 diabetic animal study* (STZ induced rats)	Alleviating morphological damage of kidney	Through reducing Ang II in plasma and kidney and inhibiting renal AT(1)R [39]
		*Cellular study* (mesangial cells)	Suppressing FN secretion	Through TGF-*β*1 way [38]

SQABC	Radix astragali and* Salvia miltiorrhiza *	*Type 1 diabetic animal study* (STZ induced rats)	Reducing 24 h UP excretion and improving reabsorption function	Through enhancing antioxidative activity and upregulating megalin [40, 41]
		*Cellular study* (NRK-52E cells)	Protecting HG injured NRK-52E cells and improving protein uptake	

TSF	*Astragalus, *raw* Rehmannia *root, sanchi root, euonymus branchlet, rhubarb, bitter orange, anddogwood fruit	*Human study* (DN patients)	Regulating and improving phospholipids metabolism Decreasing in vivo Cys, Hcy, SAM, and SAH	Through inhibiting PKC pathway and reducing phospholipids metabolism; improving in vivo hypomethylation and oxidative stress [42, 43]
		*Type 2 diabetic animal study* (db/db mice)	Upregulating JAK1, JAK2, and STAT3 and downregulating STAT4	Regulating the JAK/STAT/SOCS pathway [44]

TSL	Radix astragali, radix* Rehmannia, *leech, bile south star, Artemisia anomala, and Ze lan	*Type 1 diabetic animal study* (STZ + nephrectomized rat)	Decreasing ECM components	Through downregulating TGF-*β*1 and TIMP-2 and upregulating MMP-2 expression [45]

TXL	Scorpion, leech, Centipede, groundbeetle, Cicada, Borneol, radix paeoniae rubra, and ginseng	*Human study* (DN patients)	Improving renal function, repairing the renal tubular interstitial damage, and delaying the progression of DN	Through reducing plasma ET-1 and UAER [46]
		*Cellular study* (HKCs)	Lowering miRNA-21 expression in tissue, serum, and cells, increasing E-cadherin and decreasing *α*-SMA expression, and decreasing collagen IV and FN and increasing Ccr	Through regulating miRNA-21-induced EMT [47]
		*Type 2 diabetic animal study* (KK-Ay mice)	Reducing TGF-*β*1 and Smad3 expressions, restoring Smad7, decreasing collagen IV, FN,and 24 h UAER, BUN, and increasing Ccr	

XCHT	Radixbupleuri*, Scutellaria baicalensis *Georgi radix*, Panax ginseng, Pinellia ternata *tuber, *Glycyrrhiza glabra*, Ginger slice, and *Zizyphus vulgaris *Lam.* fructus *	*Type 1 diabetic animal study* (STZ induced rats) *Cellular study* (mesangial cells)	Decreasing the expression of TGF-*β*1, FN, and collagen IV and increasing BMP-7 expression	Through decreasing oxidative stress and productions of TGF-*β*1, FN, and collagen IV [48]

XKG	Radix astragali, Mountain* Cornus*, leech, and winged euonymus twig	*Type 1 diabetic animal study* (STZ + nephrectomized rat)	Decreasing fasting blood pressure and urinary protein in 24 hrs	Through downregulating TGF-*β*1 expression [49, 50]
		*Cellular study* (mesangial cells)	Inhibiting high glucose induced RMC proliferation	

XXD	Radix et rhizoma rhei, rhizoma coptidis, and radix* Scutellaria *	*Type 2 animal studies* (HFD + STZ induced rats, db/db mice)	Attenuating albuminuria and renal pathological changes, reducing AGEs, and inhibiting RAGE and inflammation factors expression	Through downregulating NF-*κ*B pathway and reducing renal AGEs and RAGE [51, 52]

XZT	Radix astragali, radix* Rehmannia*, fructus ligustri lucidi, *Scutellaria baicalensis *Georgi, rhizoma coptidis, dodder weed, fairy spleen, and* Salvia miltiorrhiza *	*Type 1 diabetic animal study* (STZ induced rats)	Decreasing blood glucose and HbA1C, improving renal function, ameliorating proteinuria, and reducing glomerular extracellular matrix expansion	Through inhibiting AGEs accumulation and RAGE mRNA levels renal cortex [53]

ZDP	Rhizoma anemarrhenae, cortex phellodendri, radix* Rehmannia *preparata, rhizoma* dioscorea*, fructus corni, Moutan cortex*, Alismatis *rhizoma, and* Poria *	*Type 1 diabetic animal study* (STZ induced rats)	Ameliorating DN	Through inhibiting glucose and lipid metabolism and enhancing methylamine metabolism [54]

ZHM	*Sargassum *and rhizoma rhei	*Cellular study* (human mesangial cells)	Preventing the process of DN	Through decreasing TGF-*β*1 and collagen IV expression [55]

ZQR	fructus ligustri lucidi*, eclipta Prostrata, *and* Dioscorea opposita *	*Type 2 diabetic animal study* (HFD + STZ induced rats)	Inhibiting TGF-*β*1 and FN overexpression in the renal cortex	Through inhibiting SREBP-1c overexpression and its target [17]

ZSTL	Raw* Astragalus*, *Angelica*, safflower, zedoary turmeric, dodder, radix* Rehmannia*, dogwood, *Poria*, *Epimedium*, earthworm, and* Schisandra *	*Human study* (DN patients)	Improving HbA1c and FBG, TC, TG, UAER, Scr, ANP, ET-1, and VEGF	Through modifying ANP, ET-1, and VEGF [56]

**Table 2:** Applications of single herbal TCM and/or monomers in DN.

Name	Origins	Methods	Results	Pathways

*Astragalus *	Radix astragali	*Human study* (DN patients)	Decreasing BUN, Scr, and proteinuria and improving CCr and serum albumin level	[63]
		*Type 2 diabetic animal study* (KK-Ay mice)	Increasing Smad7 expression, inhibiting TGF*β*R-1, Smad3, and its phosphorylation expression, and decreasing TGF-*β*1 mRNA level	Rebalancing TGF*β*/Smads signaling [64]
		*Cellular study* (kidney fibroblast)	Upregulating c-met expression	c-met pathway [65]

BBR	*Coptis chinensis, Hydrastis Canadensis, Berberis aristata, Berberis aquifolium*, and* Arcangelisia flava *	*Type 2 diabetic animal study* (HFD + STZ induced rats)	Suppressing histological and ultrastructural changes in kidney, improving glucose and lipid metabolism disorder, increasing cAMP, downregulating GRK2 and GRK3, and upregulating GRK6	Modulating the expression of GRKs in G protein-AC-cAMP signaling pathway [66]

Curcumin	*Curcuma longa *L.(*CLL*)	*Type 2 diabetic animal study* (db/db mice)	Decreasing albuminuria and attenuating glomerular sclerosis	Inhibiting phosphorylation of STAT3 and degradation of I*κ*B [67]
		*Cellular study *(mesangial cells)	Reducing AGE-induced oxidative stress and restoring AGE-induced mesangial cell apoptosis; Loganin inhibits FN and IL-6 expression	Reducing AGEs-induced ROS [68]

DMDD	Tuberous roots of* A. carambola *L.	*Type 2 diabetic animal study* (KK-Ay mice)	Decreasing hyperglycemia, renal AGE formation, RAGE, Scr, Ccr, and NF-*κ*B, TGF-*β*1 and enhancing reduced SOD activities	Decreasing AGEs and TGF-*β*1 levels [69]

DP	Dragon's blood	*Cellular study* (human mesangial cells)	Preventing renal fibrosis	Inhibiting SGK1 and FN expression [70]

EGB	*Ginkgo biloba *leaves	*Human study* (DN patients)	Decreasing urinary mALB, *α*1-MG, IgG, TF, RBP, and NAG	Through decreasing sICAM-1 and sVCAM-1 [71, 72]
		*Cellular study *(mesangial cells)	Suppressing MC hypertrophy and ECM accumulation	Through TGF-*β*1 and Smads pathway [73]

FA	Seeds and leaves of plants	*Type 1 diabetic animal study* (OLETF rats)	Decreasing blood glucose and urinary ACR, mesangial matrix expansion, and glomerular basement thickness	Through reducing oxidative stress and inflammation [74, 75]

Flos *A. manihot *		*Type 1 diabetic animal study* (STZ induced rats)	Preventing renal damage and podocyte apoptosis	[76]

Genipin	*Gardenia jasminoides *	*Type 1 diabetic animal study* (STZ induced mice) *Cellular study* (mouse podocyte)	Ameliorating body weight loss and urine albumin leakage, attenuating GBM thickness, suppressing upregulation of UCP2, and restoring podocin and WT1 expression	Through suppressing upregulation of mitochondrial UCP2 [77]

HCT	*Houttuynia Cordata *Thunb.	*Type 1 diabetic animal study* (STZ induced rats)	Reducing UAER, Ccr, TGF-*β*1, and collagen I and increasing BMP-7	Decreasing TGF-*β*1 and increasing BMP-7 [78]

Icariin	Herba epimedii	*Type 1 diabetic animal study* (STZ induced rats)	Relieving renal damage	Inhibiting TGF-*β*1 and Col IV expression [79]

LAB	*Salvia miltiorrhiza *	*Type 1 diabetic animal study* (STZ induced rats)	Renal MDA↓, microalbuminuria↓, mesangial expansion↓, and glomerular hypertrophy↓	TGF-*β*1 pathway [80]
		*Cellular study *(mesangial cells)	TGF-*β*1 and fibronectin secretion↓ and PKC and ROS↓	PKC and ROS pathway [80]
		*Cellular study* (VSMCs)	Inhibiting VSMCs proliferation and migration	Nrf2-ARE-NQO1 [81]

LBP	Fruit of goji berry	*Type 1 diabetic animal study* (STZ induced rats)	Increasing antioxidant enzymes and increasing scavenging oxygen radicals	Via decreased ERK 1/2 activation through PKC [82]

LGP	*Averrhoa carambola *L. (Oxalidaceae) root	*Type 1 diabetic animal study* (STZ induced mice)	Decreasing hyperglycemia, NF-*κ*B, caspase-3, caspase-8, caspase-9, and Bax expression; alleviating glomerular hypertrophy and ECM accumulation	[83]

Ligustrazine	Chuangxiong	*Human study* (DN patients)	Reducing BUN, Scr, 24 h urine protein, urine mAlb, and UAER	[84]

MC	Moutan cortex	*Type 2 diabetic animal study* (HFD + STZ induced rats)	Increasing SOD, GSH-PX, and CAT, reducing MDA; decreasing blood glucose, Scr, and urine protein and downregulating TGF-*β*2; decreasing IL-6 and MCP-1, TGF-*β*1, ICAM-1, and RAGE	Through attenuating oxidative stress and ameliorating inflammation [85–87]
		*Cellular studies* (HBZY-1 mesangial cell, rat mesangial cells)	Downregulating FN and collagen IV expression	

Morroniside	*Corni fructus *	*Type 1 diabetic animal study* (STZ induced rats)	Increasing decreased serum ALB, reducing elevated BUN, and slowing down Ccr decrease	Through inhibiting hyperglycemia and oxidative stress [88]

PNS	Radixnotoginseng	*Type 1 diabetic animal study* (STZ induced rats)	Decreasing FBG, Ccr, UAlb, and renal index	Through inhibiting VEGF and TGF-*β*1 and enhancing BMP-7 and Smad7 [89, 90]

Puerarin	*Pueraria candollei *	*Type 1 diabetic animal study* (STZ induced rats)	Decreasing collagen IV; attenuating kidney hypertrophy, mesangial expansion, and proteinuria	Downregulating MMP-9 and eNOS expression [91–93]

Rhein	Rhubarb	*Type 2 diabetic animal study* (db/db mice)	Decreasing UAE and ECM levels, decreasing TGF-*β*1 and fibronectin deposition, and decreasing hyperlipidemia	Through decreasing lipid levels [94]
		*Cellular study* (rat renal PETCs)	Inhibiting cell hypertrophy	[95]

*R. rosea *	*Rhodiola rosea *	*Type 2 diabetic animal study* (HFD + STZ induced rats)	Reducing FBG, TC, TG, Ccr, and 24 h urinary albumin	Through decreasing TGF-*β*1 expression [96]

RLM	*Rosa laevigata *Michx.	*Type 1 diabetic animal study* (STZ induced rats)	Increasing SOD activity and total antioxidant capacity, decreasing MDA and ROS levels, and inhibiting NF-*κ*B p65 and MCP-1 expression	Through regulating oxidative stress and inflammation [97]

Sequoyitol	*Aristolochia arcuata, Amentotaxus yunnanensis*, and* Crossostephium chinense *	*Type 2 diabetic animal study* (HFD + STZ induced rats)	Decreasing FBG, BUN, and Scr levels, increasing insulin and T-AOC levels in rats, and decreasing P22^phox^, P47^phox^, NF-*κ*B, and TGF-*β*1 expression in vivo and in vitro	Through glucose-lowering effects, antioxidant activity, and regulation of TGF-*β*1 expression [98]

SF	*Angelica sinensis, Lignsticum chuangxiong, Cimicifuga heracleifolia, *and other plants	*Human study* (DN patients)	Lowering UAER level and improving renal function	Through decreasing (ET) and inhibiting the combination of ET with its receptor [99]

Skimmin	*Hydrangea paniculata *	*Type 1 diabetic animal study* (STZ induced rats)	Decreasing Scr and blood glucose level, alleviating glomerular segmental sclerosis and tubular vacuolar degeneration, and downregulating TGF-*β*1 and TGF-*β*1 receptor I expression	Through inhibiting TGF-*β*1 pathway [100]

SM	*Salvia miltiorrhiza *	*Type 1 diabetic animal study* (STZ induced rats)	Decreasing TGF-*β*1, CTGF, PAI-1, FN ED-1, collagen IV, and RAGE overexpression and protecting tubular function and structure	Through inhibiting TGF-*β*1 pathway, oxidative stress, and inflammation [101–103]

TGP	*Paeonia lactiflora *Pall.	*Type 1 diabetic animal study* (STZ induced rats)	Elevating antioxidant enzyme and decreasing p-p38 MAPK and NF-*κ*B Decreasing Scr, BUN, and 24 h UP and improving renal histopathology	Through inhibiting oxidative stress [104]
		*Type 2 diabetic animal study* (HFD + STZ induced rats)		Through inhibiting Wnt/beta-catenin signaling pathway [105]

TMP	*Ligusticum chuanxiong *	*Type 1 diabetic animal study* (STZ induced rats)	Improving renal function	Through downregulating VEGF expression [106]

Triptolide	Diterpene purified from TwHF	*Type 2 diabetic animal study* (db/db mice)	Decreasing albuminuria, alleviating glomerular hypertrophy and podocyte injury, and attenuating inflammation and oxidative stress in kidney	Through inhibiting inflammation and dyslipidemia [107]
TwHF		*Human study* (DN patients)	Preventing podocyte injury	Downregulating TGF-*β*1, OPN, and CTGF [108]

VOMBP	*Magnolia biondii *Pamp.	*Type 1 diabetic animal study* (STZ induced rats)	Decreasing 24 UmAlb, sP-selectin in serum, and P-selectin in renal tissue	Inhibiting P-selectin [109]
